# Mitochondrial dysfunction associated with increased oxidative stress and α-synuclein accumulation in PARK2 iPSC-derived neurons and postmortem brain tissue

**DOI:** 10.1186/1756-6606-5-35

**Published:** 2012-10-06

**Authors:** Yoichi Imaizumi, Yohei Okada, Wado Akamatsu, Masato Koike, Naoko Kuzumaki, Hideki Hayakawa, Tomoko Nihira, Tetsuro Kobayashi, Manabu Ohyama, Shigeto Sato, Masashi Takanashi, Manabu Funayama, Akiyoshi Hirayama, Tomoyoshi Soga, Takako Hishiki, Makoto Suematsu, Takuya Yagi, Daisuke Ito, Arifumi Kosakai, Kozo Hayashi, Masanobu Shouji, Atsushi Nakanishi, Norihiro Suzuki, Yoshikuni Mizuno, Noboru Mizushima, Masayuki Amagai, Yasuo Uchiyama, Hideki Mochizuki, Nobutaka Hattori, Hideyuki Okano

**Affiliations:** 1 Department of Physiology, Keio University School of Medicine, 35 Shinanomachi, Shinjuku-ku, Tokyo 160-8582, Japan; 2 Kanrinmaru Project, Keio University School of Medicine, Tokyo, Japan; 3 Department of Cell Biology and Neuroscience, Juntendo University Graduate School of Medicine, Tokyo, Japan; 4 Department of Neurology, Kitasato University School of Medicine, Kanagawa, Japan; 5 Department of Dermatology, Keio University School of Medicine, Tokyo, Japan; 6 Department of Neurology, Juntendo University School of Medicine, Tokyo, Japan; 7 Research Institute for Diseases of Old Age, Graduate School of Medicine, Juntendo University, Tokyo, Japan; 8 Institute for Advanced Biosciences, Keio University, Yamagata, Japan; 9 Department of Biochemistry, Keio University School of Medicine, Tokyo, Japan; 10 Department of Neurology, Keio University School of Medicine, Tokyo, Japan; 11 Advanced Science Research Laboratories, Pharmaceutical Research Division, Takeda Pharmaceutical Company Limited, Kanagawa, Japan; 12 Department of Neuro-Regenerative Medicine, Kitasato University School of Medicine, Kanagawa, Japan; 13 Department of Physiology and Cell Biology, Tokyo Medical and Dental University, Tokyo, Japan; 14 Department of Neurology, Osaka University Graduate School of Medicine, Osaka, Japan

**Keywords:** Induced pluripotent stem cells, Parkinson’s disease, Parkin, Oxidative stress, Mitochondria, α-synuclein

## Abstract

**Background:**

Parkinson’s disease (PD) is a neurodegenerative disease characterized by selective degeneration of dopaminergic neurons in the substantia nigra (SN). The familial form of PD, PARK2, is caused by mutations in the *parkin* gene. *parkin*-knockout mouse models show some abnormalities, but they do not fully recapitulate the pathophysiology of human PARK2.

**Results:**

Here, we generated induced pluripotent stem cells (iPSCs) from two PARK2 patients. PARK2 iPSC-derived neurons showed increased oxidative stress and enhanced activity of the nuclear factor erythroid 2-related factor 2 (Nrf2) pathway. iPSC-derived neurons, but not fibroblasts or iPSCs, exhibited abnormal mitochondrial morphology and impaired mitochondrial homeostasis. Although PARK2 patients rarely exhibit Lewy body (LB) formation with an accumulation of α-synuclein, α-synuclein accumulation was observed in the postmortem brain of one of the donor patients. This accumulation was also seen in the iPSC-derived neurons in the same patient.

**Conclusions:**

Thus, pathogenic changes in the brain of a PARK2 patient were recapitulated using iPSC technology. These novel findings reveal mechanistic insights into the onset of PARK2 and identify novel targets for drug screening and potential modified therapies for PD.

## Background

*Parkin* is a causative gene of autosomal recessive juvenile Parkinson’s disease (PARK2). It encodes a component of an E3 ubiquitin ligase involved in mitochondrial homeostasis [1-5]. *Parkin* deficiency is thought to result in aberrant ubiquitination and compromised mitochondrial integrity, leading to neuronal dysfunction and degeneration. Several PARK2 mouse models exist, but they do not replicate all of the pathogenic changes seen in human PARK2 neurons; thus, these models do not fully account for the molecular mechanisms of PD [6-9]. A recent report demonstrated that there is a defect in dopamine (DA) utilization in PARK2 induced pluripotent stem cell (iPSC)-derived neurons [10]. However, it is not known whether neuronal homeostasis is disrupted in PARK2 patients. Furthermore, studies have yet to demonstrate whether the phenotype of PD-specific iPSC-derived neurons recapitulates the *in vivo* phenotype of the corresponding cell donor. To address these questions, we generated iPSCs from two PARK2 patients (PA and PB) [11]. In PARK2 iPSC-derived neurons, but not PARK2 fibroblasts or iPSCs, abnormal mitochondrial morphology and aberrant tubulovesicular structures adjacent to the Golgi were observed, as was increased oxidative stress. Although α-synuclein accumulation and Lewy body (LB) formation are very rare in PARK2 patients [1,12,13], we observed pathological changes and prominent LB formation, including the accumulation of α-synuclein, in postmortem brain tissue from one of the donor patients (PA). However, we obtained autopsied brain tissue from the father of donor PB, who carried the same *parkin* deletion as PB, and observed no evidence of LB formation or α-synuclein-positive cells. Consistent with these observations in postmortem brain tissue, increased α-synuclein accumulation was clearly observed in PA iPSC-derived neurons *in vitro,* but not in PB iPCS-derived neurons. These results are the first demonstration of pathogenic changes in the brain of a PARK2 patient that were recapitulated using iPSC technology. Our findings also provide mechanistic insights into PARK2 pathophysiology.

## Results & discussion

### Generation of PARK2 iPSCs

**Figure 1:**
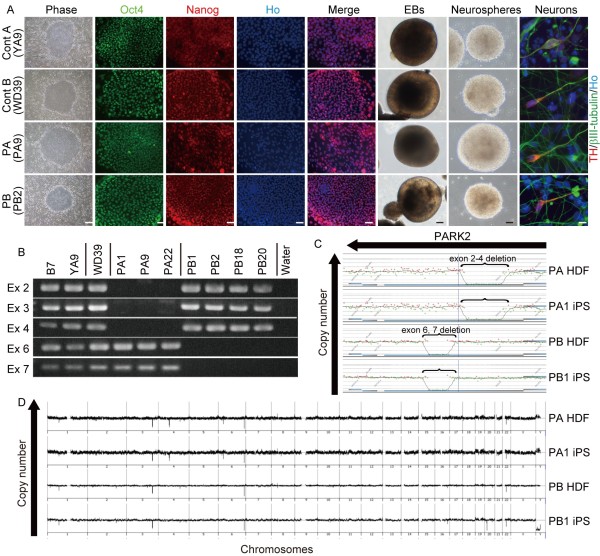
**Generation of PARK2 iPSCs. **(**A**) iPSCs derived from control and PARK2 subjects, embryoid bodies (EBs), neurospheres (NSs), and neurons. *Left three rows*: iPSCs from Control A (YA9), Control B (WD39), PA (PA9), and PB (PB2) were immunopositive for the pluripotency markers Oct4 (green) and Nanog (red). *Right three rows:* Differentiation of iPSCs into tyrosine hydroxylase (TH)-positive (red) neurons via EB and NS formation. Scale bars: Phase contrast, 400 μm; Nanog and Oct4 immunostaining, 100 μm; EBs, 25 μm; NSs, 50 μm; neurons, 10 μm. (**B**) Deletion of exons 2–4 in clones PA1, 9 and 22; and of exons 6 and 7 in clones PB1, 2, 18, and 20 was confirmed. (**C**) Exons 2–4 were deleted in human dermal fibroblasts (HDFs) from PA and in PA1 iPSC lines. Exons 6 and 7 were deleted in HDFs from PB and PB1 iPSC lines. (**D**) Copy number profiles of whole chromosomes in PARK2 HDFs and iPSCs were assessed by comparative genomic hybridization (CGH) microarray analysis; there was no evidence that genomic aberrations were introduced during the process of establishing PARK2 iPSCs.

iPSCs were generated from dermal fibroblasts isolated from two PARK2 patients carrying *parkin* mutations and two control subjects using retroviruses carrying *Oct4, Sox2, Klf4*, and *c-Myc* to reprogram the cells as previously described [14,15]. The PARK2 patients were a 71-year-old female (PA) with a homozygous deletion of *parkin* exons 2–4 and a 50-year-old male (PB) with a homozygous deletion of exons 6 and 7 (Table 1 and Additional file 1A and B). Patient PA died 1 year after enrollment in the study at the age of 72. A previously-established human iPSC clone from control subject A, 201B7 (B7), was also used [15]. In addition, the following human embryonic stem cell (hESC)-like iPSC clones were selected for detailed analysis: three controls (B7 and YA9 from control A, and WD39 from control B), three from patient PA (PA1, PA9 and PA22), and four from patient PB (PB1, PB2, PB18 and PB20) (Figure 1A and Additional file 2A and B).

**Table 1:** PA and PB patient information

	**PA patient**	**PB patient**
Race	Japanese	Japanese
Age	72 y/o	50 y/o
Sex	Female	Male
Age of onset	62 y/o	28 y/o
Mutation of *parkin*	Exon 2–4 homozygous deletions	Exon 6, 7 homozygous deletions

The PARK2 iPSCs expressed pluripotent hESC markers (Figure 1A and Additional file 2A-C) and formed teratomas containing all three germ layers (Additional file 2D). All of the retroviral transgenes were silenced in each clone (Additional file 2E). The iPSCs derived from PA and PB retained the corresponding homozygous *parkin* deletions and exhibited genomic stability (Figure 1B-D; Additional file 3A and B; and Table 1). All of the clones differentiated into neurons, including tyrosine hydroxylase (TH)-positive neurons, through a process of embryoid body and neurosphere formation (Figure 1A). Thus, all of the lines were successfully reprogrammed into a pluripotent state and were suitable for further analysis.

### Increased oxidative stress accompanied by activation of the Nrf2 pathway in PARK2 iPSC-derived neurons

**Figure 2:**
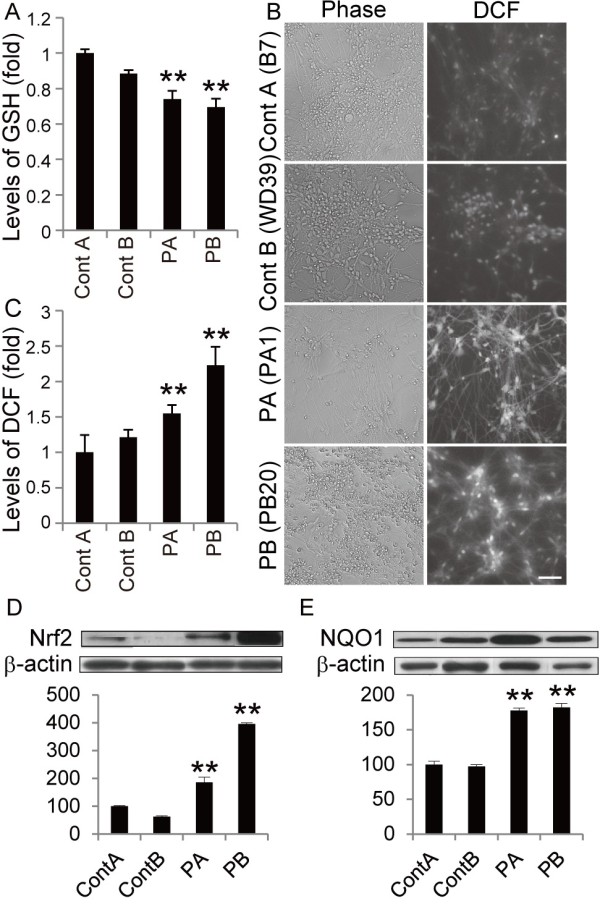
**Increased oxidative stress accompanied by activation of the Nrf2 pathway in PARK2 iPSC-derived neurons. **(**A**) GSH levels were significantly reduced in PARK2 (PA1, 9 and 22, and PB2, 18 and 20) iPSC-derived neurospheres compared with those in control A (YA9) and B (WD39) neurospheres. (**B, C**) DCF fluorescence intensity in PARK2 (PA1, 9 and 22, and PB2 and 20) iPSC-derived neurons was significantly higher than that in control A (B7) and B (WD39) neurons. (**D, E**) Immunoblot analysis of Nrf2 and NQO1 levels in iPSC-derived neurons from PA and PB. Expression of Nrf2 and NQO1 in PARK2 (PA9 and PB2) iPSC-derived neurons was significantly higher than that in control A (YA9) and B (WD39) neurons. Relative protein abundance was normalized to β-actin. ** indicates *P* < 0.01 (Mann–Whitney *U*-test). Data represent the mean and SEM of at least three experiments for each group.

Because increased levels of oxidative stress have been documented in other PD models [7,10,16,17], we examined oxidative metabolism in the iPSC clones by measuring the cellular levels of reduced glutathione (GSH). GSH reacts with reactive oxygen species (ROS) and is catalyzed by glutathione S-transferase [18]. Consistent with previous results from patient-derived cells [16], the levels of GSH in PARK2 iPSC-derived neurospheres were significantly lower than those in control iPSC-derived neurospheres (Figure 2A). We also examined ROS production using 2’, 7’-dichlorodihydrofluorescin (DCF) fluorescence to measure the levels of intracellular oxidants. The DCF fluorescence intensity in the PARK2 iPSC-derived neurons was significantly higher than that in control iPSC-derived neurons (Figure 2B and C), which indicated an increased level of oxidative stress. A recent study showed that, in PARK2 iPSC-derived neurons, monoamine oxidase (MAO)-A and -B levels and oxidative stress levels are increased, as is spontaneous DA release [10]. Here, we found no significant differences in MAO-A and -B expression levels between PARK2 and control neurons (Additional file 4A and B).

The Nrf2 pathway plays a cytoprotective role under conditions of ROS accumulation. Recent studies show that activation of the Nrf2 pathway reduces oxidative stress and provides partial protection from MPTP-mediated neurotoxicity [19]. Elevated Nrf2 expression was observed in the postmortem brain of a PD patient [20]. These data suggest a putative link between the Nrf2 pathway and PD, and prompted a closer investigation of this signaling pathway in control and PARK2 iPSC-derived neurons [19-21]. The expression of Nrf2 pathway proteins, such as Nrf2 and NADH quinone oxidoreductase (NQO1), was significantly increased in PARK2 iPSC-derived neurons (Figure 2D and E). These data are in line with previous reports [19-21], and suggest that the Nrf2 cytoprotective pathway may be activated in PARK2 iPSC-derived neurons to prevent further damage from oxidative stress. Taken together, these data demonstrated an increased level of oxidative stress accompanied by activation of the Nrf2 pathway in PARK2 neurons.

### Abnormal mitochondrial morphology and impaired mitochondrial turnover in PARK2 iPSC-derived neurons

**Figure 3:**
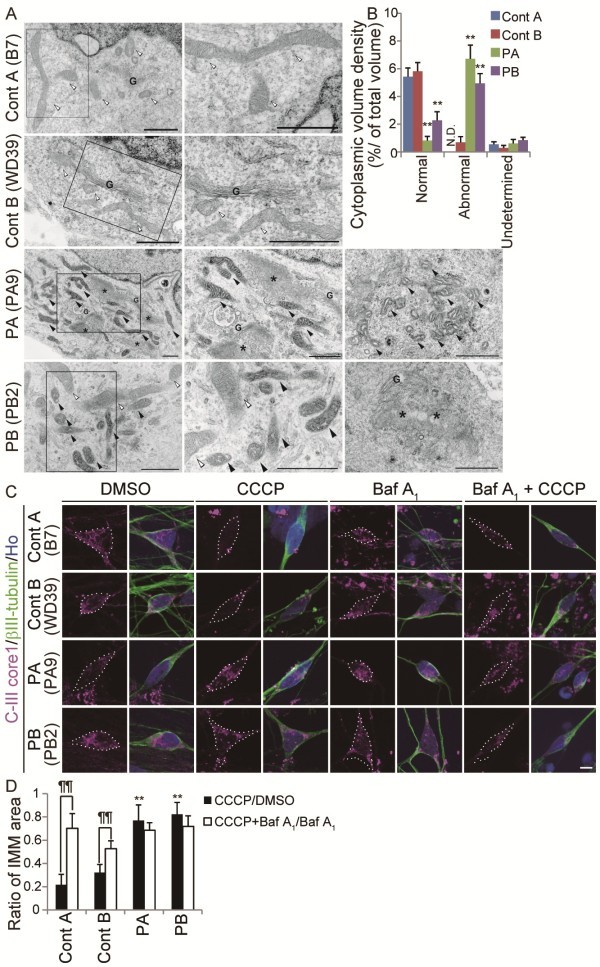
**Dysregulation of mitochondrial homeostasis in PARK2 iPSC-derived neurons.** (**A**) Electron micrographs of control A (B7), control B (WD39) and PARK2 (PA9 and PB2) iPSC-derived neurons. Boxed areas are shown in the enlarged images to the right. Control mitochondria showed a characteristically long, cylindrical profile with well-organized cristae, and the electron density of the matrix was relatively low (white arrowheads). By contrast, increased electron density of the matrix was evident in PARK2 mitochondria (black arrowheads), and the cristae often appeared swollen. As shown in PB2, some of the neurons contained both morphologically intact (white arrowheads) and abnormal (black arrowheads) mitochondria. Furthermore, abnormal tubulovesicular structures (asterisks) were observed adjacent to the Golgi cisternae (G). (**B**) The relative perikaryal volume of the abnormal mitochondria was significantly increased, and that of the normal mitochondria was decreased, in PARK2 neurons compared with control neurons. (**C**) Double labeling for the IMM marker, ComplexIII coreI (CIII-Core I; magenta) and βIII-tubulin (green) of control A (B7), control B (WD39) and PARK2 (PA9 and PB2) iPSC-derived neurons. The volume of the IMM area was reduced in control neurons treated with CCCP, but not in PARK2 neurons treated with CCCP. Administration of Baf A_1_ rescued the CCCP-induced phenotype in control neurons. (**D**) The CCCP/DMSO ratio in control A (B7 and YA9) and B (WD39) neurons was reduced after CCCP treatment. This reduction was not observed in PARK2 (PA1, 9 and 22, and PB2 and 20) iPSC-derived neurons (black bars indicate CCCP/DMSO ratio; white bars indicate Baf A_1_+CCCP/Baf A_1_ ratio). ** indicates *P* < 0.01 compared with the control; ¶¶ indicates *P* < 0.01 when comparing the black and white bars (Mann–Whitney *U*-test). At least three experiments were performed for each group, with 5–36 cells quantified per experiment. Scale bars: a, 1 μm; c, 10 μm. Error bars represent the SEM. N.D., not detected.

Increased oxidative stress (which affects anti-oxidant defense systems) and mitochondrial dysfunction are implicated in the pathogenesis of PD [1,13,21-23]. Furthermore, ROS accumulation causes both oxidative damage and mitochondrial dysfunction in the substantia nigra (SN) of *parkin*-deficient mice [7]. However, the exact mechanism of mitochondrial pathogenesis associated with PARK2 is controversial. For example, while *Drosophila parkin* mutants show abnormal mitochondrial morphology, *parkin-*knockout mice do not [7,24]. In addition, while a greater degree of mitochondrial branching is observed in fibroblasts derived from PARK2 patients, the detailed morphology of the mitochondria in these cells has not been characterized [25]. To investigate these mitochondrial abnormalities in more depth, we performed a detailed morphological analysis of mitochondria in PARK2 iPSC-derived neurons using electron microscopy. Mitochondria in PARK2 neurons from both patients showed a highly electron-dense matrix and swollen mitochondrial cristae within the inner mitochondrial membrane (IMM) (Figure 3A, black arrowheads). The perikaryal volume density of the abnormal mitochondria was significantly increased in PA and PB iPSC-derived neurons relative to control clones (Figure 3B). Furthermore, the density of normal mitochondria decreased (Figure 3B). Importantly, both abnormal and normal mitochondria were observed in PARK2 neurons (Figure 3A, white arrowheads). Abnormal mitochondria were observed in 87.8% of iPSC-derived neurons from PA, and 79.5% of iPSC-derived neurons from PB. These data indicated that abnormal mitochondrial morphology was a feature of most PARK2 iPSC-derived neurons from these patients. In addition, abnormal tubulovesicular structures were observed adjacent to the Golgi cisternae in PARK2 iPSC-derived neurons (Figure 3A). These abnormal mitochondrial and tubulovesicular structures were not observed in PARK2 fibroblasts or in undifferentiated iPSCs (Additional file 5A and B). These histological abnormalities represent novel PARK2-related neuronal pathologies.

PARKIN is involved in the mitochondrial fission/fusion system and is recruited to depolarized mitochondria to promote mitophagy [5,26-29]. In iPSC-derived neurons containing a mutation in PINK1 (a protein kinase upstream of PARKIN), PARKIN is not recruited appropriately to mitochondria [30]. We hypothesized that PARKIN-deficient human neurons would show aberrant removal of depolarized mitochondria. To examine the turnover of damaged mitochondria, we treated iPSC-derived neurons with carbonyl cyanide m-chlorophenyl hydrazine (CCCP), which triggers the loss of mitochondrial membrane potential and results in the removal of damaged mitochondria. The intensity of TMRE, a mitochondrial membrane potential-dependent dye, clearly decreased in both control and PARK2 iPSC-derived neurons treated with CCCP, which indicated a reduced mitochondrial membrane potential in both sets of neurons (Additional file 6). To determine the extent to which the damaged mitochondria were eliminated, we measured the area of the IMM after CCCP treatment. Compared with untreated cells, there was a dramatic loss of IMM area in the treated control neurons, but not in the treated PARK2 neurons (Figure 3C, left four columns; Figure 3D, black bars). To assess whether lysosomes were involved in the CCCP-induced elimination of mitochondria, we treated cells with Bafilomycin (Baf) A_1_, an inhibitor of the vacuolar type H(+)-ATPase. Baf A_1_ attenuated the CCCP-dependent reduction in the IMM area in control neurons (Figure 3C, right four columns; Figure 3D, white bars). To confirm that the abnormal turnover of damaged mitochondria was characteristic of neuronal cells, PARK2 fibroblasts and undifferentiated iPSCs were treated with CCCP. CCCP-treated PARK2 fibroblasts and undifferentiated iPSCs exhibited the same mitochondrial dynamics as CCCP-treated control cells (Additional file 5C-E). Together, these data indicated aberrant degradation of mitochondria damaged by CCCP treatment in PARK2 iPSC-derived neurons.

These results support a recently proposed working model for PD, in which damaged mitochondria accumulate due to a disruption in PARKIN-mediated mitochondrial quality control [28]. The electron microscopy data, which showed a mixture of abnormal and normal mitochondria, indicated that PARKIN-mediated mitochondrial quality control is compromised, even in young PARK2 iPSC-derived neurons. In these cells, residual normal mitochondria may have compensated for the damaged ones. Thus, while our findings suggest that the PARKIN-dependent mechanisms that regulate mitochondrial homeostasis are disrupted in PARK2 cells, further detailed analyses are required to fully understand the mechanism underlying this disruption and the implications for PD.

### Patient-specific accumulation of α-synuclein in PARK2 iPSC-derived neurons and its correlation with LB formation

LBs are pathological neuronal inclusions composed principally of α-synuclein. They are typically associated with PD and certain forms of dementia [1,13,31]. Although LBs are generally thought to be absent from PARK2 patients [1,13,31], rare cases of LB formation in the brains of PARK2 patients have been reported recently [12,32,33]. The PARKIN protein co-localizes with LBs in some patients with sporadic PD [34], and a functional interaction between PARKIN and α-synuclein is indicated by both *in vitro* and *in vivo* findings [35-37]. These results suggest that PARKIN-pathway may contribute to LB formation in PD patients.

**Figure 4:**
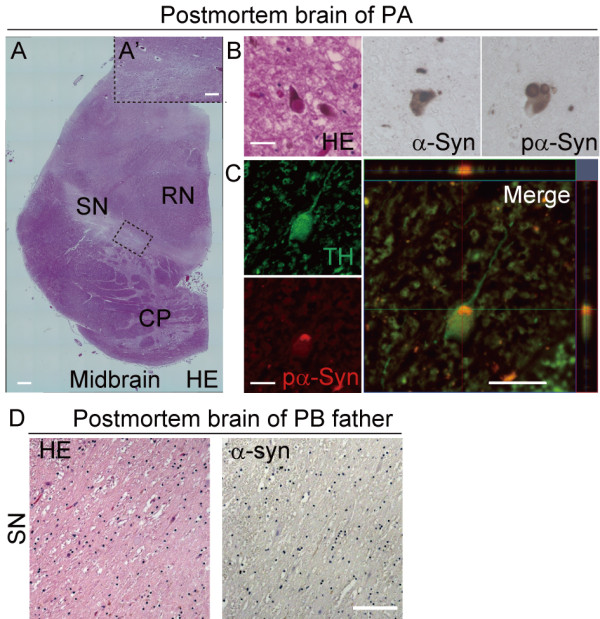
**Accumulation of LBs in the postmortem brain of patient PA. **(**A**–**C**) Immunohistochemical staining of postmortem brain tissue from patient PA. (**A**) Low magnification image of a midbrain section stained with hematoxylin and eosin (H&E). (**A’**) High magnification image of the boxed area. Melanin levels were reduced in most of the substantia nigra (SN). (**B**) (Left) High magnification image of a midbrain section stained with H&E showing the presence of Lewy bodies (LBs) in the SN. (Middle and Right) α-synuclein-positive and pα-synuclein-positive cells in the SN. (**C**) Confocal microscopy image of a TH (green) and pα-synuclein (red) double-positive SN neuron and a projected merged image: pα-synuclein accumulated in the TH-positive neuron. (**D**) Melanin levels were reduced in most of the SN. No LBs or α-synuclein-positive neurons were observed in postmortem brain tissue from the father of patient PB. Scale bars: A, 1000 μm; A’, 350 μm; B, C, 50 μm; D, 100 μm.

We were able to conduct a histopathological analysis of postmortem brain tissue from patient PA. Hematoxylin and eosin staining of the SN revealed low levels of brown-black melanin pigment compared with healthy SN tissue (Figure 4A and A’). Surprisingly, LBs accumulated in the SN and other areas of the brain in patient PA (Figure 4B and Table 2). Furthermore, α-synuclein and pα-synuclein immunoreactive puncta and neurites were observed in the areas where LBs were present (Figure 4B). TH/pα-synuclein double-positive neurons were also detected in the SN (Figure 4C). Of note, α-synuclein-positive/TH-negative or pα-synuclein-positive/TH-negative neurons in the SN and other areas of the brain tissue from patient PA’s brain were observed (Table 2). These data suggested that α-synuclein accumulated not only in TH+ neurons, but also in other types of neurons. Postmortem tissue from the brain of the father of patient PB was also examined. The father carried a homozygous deletion of exons 6 and 7 of the *parkin* gene (Figure 1B, Additional file 1B and 7A), similar to patient PB. There was no evidence of LBs or α-synuclein-positive neurons in the autopsied brain tissue of the father (Figure 4D). Thus, since the genetic background of patient PB and his father are likely to be very close (Additional file 1B and 7A), these results are probably reflective of a specific phenotype of patient PB, which was different from that in patient PA (Figure 4A-D).

**Table 2:** LB type pathology in PA patient’s postmortem brain

**Brain area**		**LB type pathology**
Brainstem lesion	IX-X	+++
	LC	+++
	SN	++
Basal forebrain/Limbic	nbM	++
	Amy	++
	Ent	+
	Cing	+
Neocortical	T	-
	F	-
	P	-

IX-X, motor cranial nerves IX-X; *LC*, Locus Coeruleus; *SN*, Substantia Nigra; *nbM*, nucleus basal of Meynert; Amy, Amygdala; Ent, Entorhinal cortex; T, Temporal lobe; F, Frontal lobe; P, Parietal lobe.

**Figure 5:**
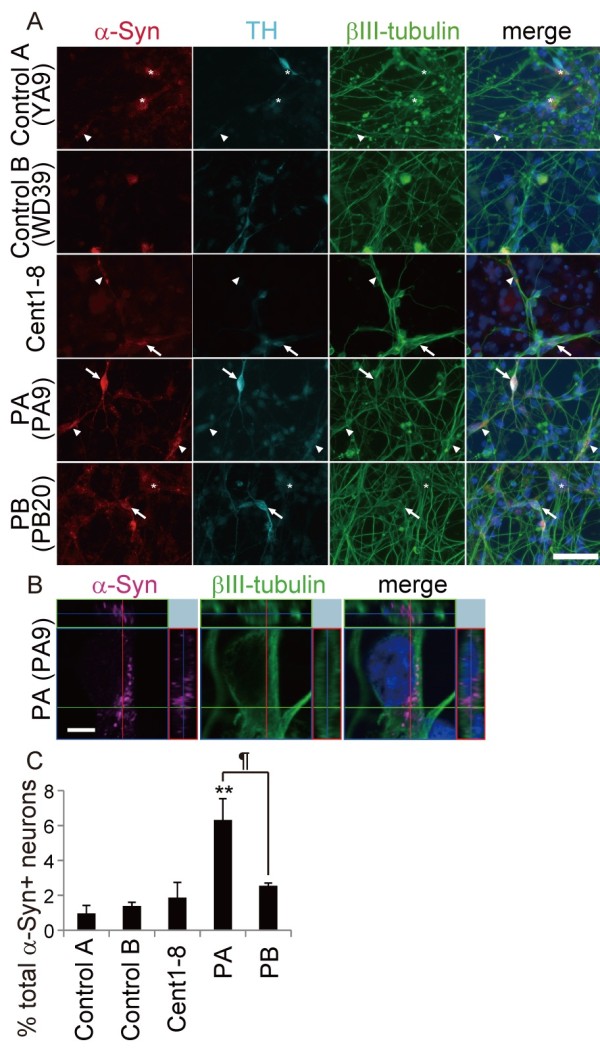
**α-synuclein accumulation in PARK2 iPSC-derived neurons. **(**A**–**C**) Triple labeling for α-synuclein (red), tyrosine hydroxylase (TH; cyan), and βIII-tubulin (green) along with Hoechst (blue) staining of control A (B7), control B (WD39), Cent1-8, and PARK2 (PA9 and PB20) iPSC-derived neurons. (**A**) Arrows indicate α-synuclein+/TH+/βIII-tubulin+ neurons; arrowheads indicate α-synuclein+/TH-/βIII-tubulin+ neurons. Note the presence of α-synuclein+/TH-/βIII-tubulin- non-neural cells (asterisks). (**B**) High magnification confocal projection image of an α-synuclein (magenta)/βIII-tubulin (green) double-positive PA9 iPSC-derived neuron. (**C**) The proportion of α-synuclein+/βIII-tubulin+ neurons relative to βIII-tubulin-positive neurons was significantly higher in PA (PA1, 9 and 22) iPSC-derived neurons than in control A (B7 and YA9), control B (WD39) and Cent1-8 iPSC-derived neurons. Scale bars: A, 50 μm; C, 5 μm. ** indicates *P* < 0.01; * and ¶ indicate *P* < 0.05 (Mann–Whitney *U*-test). Data represent the mean and SEM of at least three experiments for each group.

To determine whether iPSC-derived neurons recapitulated the *in vivo* phenotypes of the corresponding cell donors, we next examined α-synuclein accumulation in PARK2 iPSC-derived neurons. To rule out the possibility that α-synuclein expression in undifferentiated PARK2 iPSCs was increased by multiplication of the *SNCA* gene, genomic aberrations acquired during the process of iPSC establishment, or by repeated passage of the cells, the *SNCA* gene copy number in iPSCs was quantified by genomic qPCR. A comparison with control iPSCs showed that iPSCs from both PA and PB carried the normal number of *SNCA* gene copies (Additional file 8A). Moreover, immunostaining for α-synuclein did not reveal any increase or decrease in α-synuclein protein levels in PARK2 iPSCs (Additional file 8B). As a control for LB formation, we generated iPSC-derived neurons from a 106-year-old woman (designated Cent1-8), since previous work suggested that aging is a predisposing factor for LB formation in PD patients [31,38]. Since α-synuclein was also expressed in non-neural cells, triple labeling for α-synuclein, βIII-tubulin, and TH was performed to ensure that only neurons were examined (Figure 5A, asterisks). The proportion of α-synuclein-positive iPSC-derived neurons that were also positive for βIII-tubulin from PB was similar to that in the controls (including Cent1-8); however, the proportion was significantly higher in PA. These results were consistent with the *in vivo* phenotypes of the cell donors based on analysis of postmortem brain tissue (1629, 357, 805, 3747, and 4330 iPSC-derived βIII-tubulin+ neurons in control A, control B, Cent1-8, PA and PB respectively; Figure 5A-C, arrows and arrowheads). Thus, the increase in α-synuclein expression levels seen in PARK2 iPSC-derived neurons cannot be attributed solely to the effects of aging, but associated with the disease phenotype.

The obvious LB-formation was observed in the postmortem brain of PA patient, who showed a late onset at 61 years, corresponding to the enhanced α-synuclein accumulation in the iPSC-derived neurons from the same patient. Thus, it is likely that early-stage LB formation was recapitulated *in vitro* in iPSC-derived neurons. Furthermore, the present findings are consistent with recent work by several groups, which suggest that the age of onset of PARK2 in patients with LB formation (41 on average) is later than in patients without LB formation (below 40) [12,32,33]. The earlier onset in patient PB (at 28 years) than in PA (at 61 years) would be consistent with the finding of lower α-synuclein accumulation in PB iPSC-derived neurons compared with PA iPSC-derived neurons. On the other hand, and in contrast to the observations of brain tissue from PA, analysis of brain tissue from the father of patient PB, in whom the onset of PD was 39 years of age, revealed no evidence of LB formation (Figure 4D). Importantly, PA iPSC-derived neurons showed significantly more α-synuclein accumulation than PB iPSC-derived neurons (Figure 5A and C). These results suggest that the extent of α-synuclein accumulation is an important factor in LB formation. Then, how can we explain the difference of α-synuclein accumulation between PA and PB patients-derived neuronal cells? It is possible that PA is a rare example of PARK2 complicated by sporadic PD. Although both PA and PB iPSCs showed a normal *SNCA* gene copy number, it is possible that PA-derived cells acquired an unknown gene mutation relating to LB formation. Thus, we cannot rule out the possibility that other factors may affect LB formation in PARK2 patients. Further analyses will be required to identify these putative factors. Although iPSC clones from sporadic and familial PD patients were recently established [17,30,39-42], this report is the first to demonstrate that the phenotype of PD-specific iPSC-derived neurons replicates the *in vivo* phenotype seen in postmortem brain tissue from the corresponding cell donor.

## Conclusions

In summary, dysfunctional neuronal homeostasis (characterized by increased oxidative stress and activation of the Nrf2 pathway), impaired mitochondrial function, and increased α-synuclein accumulation were observed in PARK2 iPSC-derived neurons. These results indicate that PARK2-associated phenotypes may appear soon after, or possibly even before, the onset of PARK2. Detailed analyses of PARK2 iPSC-derived neurons, particularly mature neurons, to determine the time course of LB accumulation and synaptic dysfunction will be of great interest. Such analyses will further our understanding of the pathogenesis of PARK2 as well as sporadic PD. The ultimate goal is the development and application of novel preventative therapies for PD.

## Materials & methods

### Isolation of human skin fibroblasts and generation of iPSCs

For control A, human dermal fibroblasts (HDFs) from the facial dermis of a 36-year-old Caucasian female (Cell Applications Inc.) were used to establish iPSCs (201B7; Passage 20–29, YA9; Passage 15–24). The 201B7 iPSCs were kindly provided by Dr. Yamanaka [15]. A skin-punch biopsy from a healthy 16-year-old Japanese female obtained after written informed consent (Keio University School of Medicine) was used to generate the control B iPSCs (WD39; Passage 8–17). PA iPSCs (PA1, 9, and 22; Passage 10–19) and PB iPSCs (PB1, 2, 18, and 20; Passage 8–17) were generated from a 71-year-old Japanese female patient and a 50-year-old Japanese male patient, respectively, using the same methods used to generate control B iPSCs. The maintenance of HDFs, lentiviral production, retroviral production, infection, stem cell culture and characterization, and teratoma formation were performed as described previously [14,15]. All of the experimental procedures for skin biopsy and iPS production were approved by the Keio University School of Medicine Ethics committee (Approval Number: 20-16-18) and Juntendo University School of Medicine Ethics committee (Approval Number: 2012068). hESCs (KhES-1; Passage 29–38 (kindly provided by Dr. Norio Nakatsuji) were cultured on feeder cells in iPS culture media [43].

### *In vitro* differentiation of human iPSCs

Neural differentiation of iPSCs was performed as previously described [44] with slight modifications (Okada et al., manuscript in preparation). Briefly, iPSC colonies were detached from feeder layers and cultured in suspension as EBs for about 30 days in bacteriological dishes. EBs were then enzymatically dissociated into single cells and the dissociated cells cultured in suspension in serum-free media (MHM) [44] for 10 to 14 days to allow the formation of neurospheres. Neurospheres were passaged repeatedly by dissociation into single cells followed by culture in the same manner. Typically, neurospheres between passages 3 and 8 were used for analysis. For terminal differentiation, dissociated or undissociated neurospheres were allowed to adhere to poly-L-ornithine- and fibronectin-coated coverslips and cultured for 10 days.

### Immunocytochemical analysis of iPSCs and neurons

For immunocytochemical analysis, cells were fixed with phosphate buffered saline (PBS) containing 4% paraformaldehyde (PFA) for 30 min at room temperature (RT). The cells were analyzed by immunofluorescence staining using antibodies to the following proteins: β-III-tubulin (1:1000, Sigma), NANOG (1:100, ReproCELL), OCT3/4 (1:200, Santa Cruz Biotechnology), SSEA-4 (1:200, Millipore), TRA-1-60 (1:200, Millipore), TH (1:100, Millipore), α-synuclein (1:500, Invitrogen), pα-synuclein (1:1000, Wako), cleaved-Caspase3 (1:500, Cell Signaling) and ComplexIII (C-III)-core I (1:200, Invitrogen). Cells were washed with PBS after incubation with the primary antibody, followed by incubation with an Alexa Fluor 488-, Alexa Fluor 555-, or Alexa Fluor 647-conjugated secondary antibody (1:500, Invitrogen). Images were obtained using Apotome (Zeiss) or LSM-710 confocal (Zeiss) microscopes.

### PCR amplification of genomic DNA

Genomic DNA was purified from HDFs and iPSCs using a DNeasy kit (Qiagen). The PCR conditions used have been previously described [2,42].

### Reverse transcription (RT)-PCR

RNA isolation and reverse transcription (RT)-PCR were performed as previously described [44]. The amount of cDNA was normalized to *β-actin* mRNA. Real-time RT-PCR was performed on a ABI PRISM Sequence detection System 7900HT (Applied BioSystems) using SYBR premix ExTaq (Takara). Primers for the detection of Oct4, the transgenes *Oct4-tg, Sox2-tg, Klf4-tg* and *c-Myc-tg*, and MAO-A, and -B have been previously described [10,15].

### Teratoma assay

To assess teratoma formation, iPSCs were injected into the testis of 8-week-old NOD/SCID mice (OYG International) as previously described [14]. Eight weeks after transplantation, tumors were dissected and fixed with 4% PFA in PBS. Paraffin-embedded tissue was sectioned and stained with H&E. Images were obtained using a BZ-9000 (Keyence) microscope.

### CGH array

Genomic DNA was restricted, labeled, and purified using the Agilent Oligo CGH Microarray Kit (Agilent Technologies) according to the manufacturer’s protocol. Labeled genomic DNA was processed for hybridization on a 4x 180K microarray (Agilent Technologies). Processing was performed as instructed by the manufacturer. The genomic analysis was performed using Agilent Genomic Workbench ver. 6.0 software (Agilent Technologies).

### Metabolism assays

Reduced GSH levels were measured according to the kit manufacturer’s protocol (GSH-Glo Glutathione Assay; Promega). Chymotrypsin-like proteasome activity was measured using a Cell-Based Proteasome-Glo Assay according to the manufacturer’s instructions (Promega). Briefly, neural cells (1.0 × 10^4^) derived from neurospheres were seeded in triplicate into a white 96-well plate (Nunc). Prepared reagent (100 μl) was added to each well. After incubation for 10 min at RT, luminescence intensity was recorded. ROS levels were determined by measuring DCFH-DA fluorescence (Invitrogen). Briefly, neurons were incubated with 5 μM DCFH-DA and Hoechst (1:2000) for 30 min at 37°C, after which they were washed with PBS and then incubated in differentiation media. Fluorescence was measured by an In Cell Analyzer 2000 system (GE Healthcare Biosciences).

### Protein analysis

Differentiated neurons were harvested in MAPK lysis buffer containing proteinase inhibitor, and protein concentrations were measured by BCA assay (Thermo Scientific). Samples were diluted to yield equivalent protein concentrations and then 4 μg was denatured by the addition of 4X sample buffer (Invitrogen) supplemented with β-mercaptoethanol followed by boiling. Samples (7 μl/lane) were loaded onto a 4–20% SDS-polyacrylamide gradient gel. Membranes were incubated in blocking solution with the indicated primary antibodies at 4°C overnight. Immunoreactive proteins were detected with horseradish peroxidase (HRP)-conjugated secondary antibodies and then visualized by chemiluminescence (Pierce, Rockford, IL, USA) according to the manufacturer’s instructions. Quantification of band intensities was performed using an RAS4000 system. The primary antibodies used were anti-NQO1 (1:1000, Abcam), anti-NRF2 (1:1000, Santa Cruz Biotechnology) and β-actin (1:5000, Cell Signaling).

### CCCP and Baf A_1_ treatments

Neurons were cultured with 30 μM CCCP (Sigma-Aldrich) or DMSO, with or without 5 μM Baf A_1_ (Sigma-Aldrich), for 48 h. The cells were then fixed and stained for βIII-tubulin and C-III Core I, and counterstained with Hoechst. To quantify the IMM area of the neurons, the cytoplasmic area was extracted as shown in Figure 3C. The C-III Core I-positive signals within the extracted area were then converted to gray-scale and digitized. The IMM area was quantified from the digitized values using Image J software.

### Tetramethylrhodamine ethyl ester (TMRE) staining

iPSC-derived neurons were incubated with 1nM TMRE (Invitrogen) for 15 min at 37°C and then observed under an Olympus IX81 microscope.

### Electron microscopy

Cells were fixed with 2% glutaraldehyde/2% PFA in 0.1 M phosphate buffer (PB) (pH7.2), post-fixed with 1% OsO4 in 0.1 M PB (pH 7.2), blocked and stained with a 2% aqueous solution of uranyl acetate, dehydrated with a graded series of ethanol, and then embedded in Epon 812 (TAAB). Coverslips were detached and the embedded samples were placed under a stereomicroscope to identify the cells of interest. Ultrathin sections were cut with a Leica UC6 or UC7 ultramicrotome (Leica Microsystems) and then stained with uranyl acetate and lead citrate. Samples were observed with a Hitachi H7100 or HT7700 electron microscope.

### Morphometry

Morphometric analysis was used to measure the volume density of mitochondria in the neuronal perikarya as previously described [45]. Briefly, electron micrographs of neurons (n = 20, 23, 41, and 44 for control A (B7), control B (WD39), PA9 and PB2, respectively) were obtained at a magnification of ×7000. After enlarging to three times the original magnification, point-counting was carried out to determine the volume density using a double-lattice test system with 1.5 cm spacing. Mitochondria were classified as normal, abnormal, or undetermined. The abnormal mitochondria were defined as those with irregularly arranged cristae, or with a high electron-dense matrix. The volume density (Vv) of each type of mitochondrion was expressed as percent volume according to the following formula: Vv = (Pi/Pt) × 100 (%), where Pi is the number of points falling on each mitochondrial structure and Pt is the number of points falling on the neuronal perikarya.

### Immunohistochemical analysis of autopsied brain tissue

The ethical committee of the Kitasato University School of Medicine and Juntendo University School of Medicine reviewed and approved the protocol for analysis of autopsied brain tissue. Patients and control subjects were informed of the study and gave written informed consent. Brain tissue from patient PA was obtained following her death at age 72; brain tissue from the father of patient PB was obtained when he died at age 70 [46]. Tissue was fixed with 10% formalin and then embedded in paraffin. Midbrain sections (6 μm thick) were cut, deparaffinized with xylene, and then rehydrated in ethanol. After being boiled and treated with H_2_O_2_, sections were subjected to immunofluorescence staining with antibodies to the following proteins: α-synuclein (1:500, Invitrogen), pα-synuclein (1:1000, Wako), and TH (1:1000, Calbiochem). After washing with PBS, sections were incubated with a biotinylated secondary antibody (1:500; Vector Laboratories Inc.) at RT for 1 hr followed by incubation with an avidin-biotin peroxidase complex (Vector Laboratories Inc.) for 1 hr. Immunoreactive proteins were visualized using 3,3-diaminobenzidine (DAB; Wako Pure Chemical Industries) and nuclear fast red staining. For immunofluorescence, FITC-conjugated and Cy3-conjugated secondary antibodies (1:500; Jackson Immunoresearch Laboratories) were used. Images were obtained using a BIOREVO (Keyence) and a confocal laser-scanning LSM710 (Zeiss) microscope.

### Statistical analysis

Values represent the mean ± SEM. The Mann–Whitney *U*-test was used to evaluate differences between groups. A *P* value of < 0.05 was considered significant.

## Competing interests

The authors declare that they have no competing interests.

## Authors' contributions

YI, YO, WA, and HO conceived and designed the experiments. YI performed most of the experiments, analyzed data, and wrote the manuscript. YO, and HO edited the manuscript. YO developed the quality control system, neural differentiation method for the iPSCs and performed CGH microarray data analysis. WA generated the WD39 iPSCs. NK, KH, MS and AN performed western blotting analysis. TN performed some parts of *in vitro* culture assay. SS, MF, YM, HM and NH examined and recruited PARK2 patients. TK, MO, and MA performed biopsies and established the skin fibroblasts. AH, TS, TH and MS performed preliminary experiments for the metabolome analysis. TY, DI, AK and NS provided cent8-1 iPSCs. YI and NM designed the CCCP treatment experiment. MK and YU performed the electron microscopic analysis. HH, MT, HM and NH performed the histopathological studies of the postmortem brain of PA. All authors read and approved the final manuscript.

## Supplementary Material

Additional file 1 **Genetic studies of family.** (A) An arrow indicates PA patient. (B) An arrow indicates PB patient. Filled circles and squares, women and men with PARK2 mutation; Open circles and squares, normal women and men; Diamond shapes, family members whose DNA samples were not analyzed. Symbols with lines through them represent the deceased. Click here for file

Additional file 2 **Characterization of control and PARK2 iPSCs.** (A) Control A (YA9), Control B (WD39), PA (PA9), and PB (PB2) iPSCs expressed the pluripotency markers SSEA4 (red) and TRA1-60 (green). Scale bar, 100 μm. (B) iPSCs established from patients PA (PA1, PA22) and PB (PB1, PB18, and PB20) were positive for the pluripotency markers Nanog (red), Oct4 (green), SSEA4 (red), and TRA1-60 (green). Scale bars: phase images, 200 μm; immunofluorescence images, 100 μm. (C) Levels of endogenous *Oct4* mRNA in the generated iPSCs were similar to those in KhES1 cells, a human embryonic stem cell (hESC) line [42]. Expression levels were normalized to that of KhES1 (set as 1). (D) Cont A (YA9), Cont B (WD39), PA (PA1, 9 and 22), and PB (PB1, 2, 18 and 20) iPSCs gave rise to teratomas with all three germ layers, confirming pluripotency. Scale bar, 100 μm. (E) Silencing of transgenes in control and PARK2 iPSC clones. Expression levels were normalized to the positive control of fibroblasts in cultures assayed 6 days after retroviral infection ( = 100). Cont A, Control A; Cont B, Control B. Click here for file

Additional file 3 **Confirmation of*parkin*deletions and genomic stability of PARK2 iPSCs using comparative genomic hybridization (CGH) microarray analysis.** (A) Exons 2–4 were deleted in the PA9 and PA22 iPSC lines. Exons 6 and 7 were deleted in the PB2, 18, and 20 iPSC lines. (B) Copy number profiles of whole chromosomes in PARK2 iPSCs assessed by CGH microarray analysis revealed that no genomic aberrations were introduced during the process of establishing PARK2 iPSCs. Click here for file

Additional file 4 **Expression level of MAO-A and -B showed no difference among Control and PARK2 iPSC-derived neurons.** (A,B) qRT-PCR measurement of MAO-A and -B transcripts in PARK2 (PA (1, 9 and 22) and PB (1, 2 and 20)) iPSC-derived neurons showed no difference compared to those in Cont A (B7 and YA9). ContA; Control A, ContB; Control B. Click here for file

Additional file 5 **Healthy mitochondria in PARK2 fibroblasts and iPSCs.** (A, B) Electron micrographs of fibroblasts (upper panels) and iPSCs (lower panels) from Control (Cont A and Cont B) and PARK2 patients (PA and PB). Mitochondria in the fibroblasts and iPSCs from both groups showed long, cylindrical profiles with well-organized cristae, and the electron density of the matrix was relatively low (asterisks). Scale bar, 0.25 μm. Cont A, Control A; Cont B, Control B. (C) Fibroblasts were treated with 30μM CCCP or DMSO for 48 h, followed by staining for CIII coreI (magenta) to label the internal mitochondrial membrane (IMM) and counterstaining with Hoechst (Ho, blue). Mitochondrial size decreased after CCCP treatment in both Control (Cont A and Cont B) and PARK2 (PA and PB) fibroblasts. Scale bar, 20 μm. (D) iPSCs were treated with 30 μM CCCP or DMSO for 48 h and then stained for CIII coreI (magenta) to label IMM, Oct4 (blue) to label iPSCs, and Hoechst (Ho, white). Mitochondrial size in Control (Cont A (B7), Cont B (WD39)), and PARK2 (PA9 and PB2) iPSCs decreased after CCCP treatment. Scale bar, 20 μm (E) CCCP/DMSO ratios in Control (Cont A (B7, YA9), Cont B (WD39)), and PARK2 (PA9 and 22 and PB2 and 20) iPSCs (Mann Whitney *U*-test). Data represent the mean and SEM (*n* > 3 for each group). Click here for file

Additional file 6 **Mitochondrial membrane potential after CCCP treatment in control and PARK2 iPSC-derived neurons.** (A) iPSC-derived neurons were treated with 30 μM CCCP or DMSO for 48 h, after which they were stained for the mitochondrial membrane potential marker, TMRE. The intensity of TMRE (yellow) was clearly reduced in control (Cont A (B7), Cont B (WD39)), and PARK2 (PA9 and PB2) iPSC-derived neurons. Scale bar, 50 μm. Click here for file

Additional file 7 **Confirmation of*parkin*deletions carried by the father of patient PB.** (A) Deletion of exons 6 and 7 was confirmed in blood samples from PB and the father of PB by PCR. Click here for file

Additional file 8 **α-Synuclein signals are not seen in PARK2 iPSCs.** (A) Quantitative genomic PCR analysis for SNCA exons 1 and 4 demonstrated a normal copy number in PARK2 (PA1, 9 and 22, and PB1, 2, 18 and 20) iPSCs. The copy number was the same as that observed for Cont A (B7 and YA9) and Cont B (WD39). The *SNCA* gene copy number was normalized to β-globin *(HBB)* and β2-microglobulin *(B2MG).* (B) iPSCs were stained for α-synuclein (red), Oct4 (green; to label iPSCs) and Hoechst (blue). No α-synuclein signals were observed in Cont A (B7 and YA9), Cont B (WD39), or PARK2 (PA9 and 22, PB2 and 20) iPSCs. Scale bar, 50 μm. Click here for file

## References

[B1] Farrer MJ Genetics of Parkinson disease: paradigm shifts and future prospects Nature reviews 2006 7 306 318 10.1038/nrg1831 16543934

[B2] Kitada T Asakawa S Hattori N Matsumine H Yamamura Y Minoshima S Yokochi M Mizuno Y Shimizu N Mutations in the parkin gene cause autosomal recessive juvenile parkinsonism Nature 1998 392 605 608 10.1038/33416 9560156

[B3] Shimura H Hattori N Kubo S Mizuno Y Asakawa S Minoshima S Shimizu N Iwai K Chiba T Tanaka K Suzuki T Familial Parkinson disease gene product, parkin, is a ubiquitin-protein ligase Nat Genet 2000 25 302 305 10.1038/77060 10888878

[B4] Whitworth AJ Pallanck LJ The PINK1/Parkin pathway: a mitochondrial quality control system? J Bioenerg Biomembr 2009 41 499 503 10.1007/s10863-009-9253-3 19967438

[B5] Youle RJ Narendra DP Mechanisms of mitophagy Nat Rev Mol Cell Biol 2011 12 9 14 10.1038/nrm3028 21179058 PMC4780047

[B6] Goldberg MS Fleming SM Palacino JJ Cepeda C Lam HA Bhatnagar A Meloni EG Wu N Ackerson LC Klapstein GJ Parkin-deficient mice exhibit nigrostriatal deficits but not loss of dopaminergic neurons J Biol Chem 2003 278 43628 43635 10.1074/jbc.M308947200 12930822

[B7] Palacino JJ Sagi D Goldberg MS Krauss S Motz C Wacker M Klose J Shen J Mitochondrial dysfunction and oxidative damage in parkin-deficient mice J Biol Chem 2004 279 18614 18622 10.1074/jbc.M401135200 14985362

[B8] Perez FA Palmiter RD Parkin-deficient mice are not a robust model of parkinsonism Proc Natl Acad Sci USA 2005 102 2174 2179 10.1073/pnas.0409598102 15684050 PMC548311

[B9] Sato S Chiba T Nishiyama S Kakiuchi T Tsukada H Hatano T Fukuda T Yasoshima Y Kai N Kobayashi K Decline of striatal dopamine release in parkin-deficient mice shown by ex vivo autoradiography J Neurosci Res 2006 84 1350 1357 10.1002/jnr.21032 16941649

[B10] Jiang H Ren Y Yuen EY Zhong P Ghaedi M Hu Z Azabdaftari G Nakaso K Yan Z Feng J Parkin controls dopamine utilization in human midbrain dopaminergic neurons derived from induced pluripotent stem cells Nat Commun 2012 3 668 22314364 10.1038/ncomms1669 PMC3498452

[B11] Mattis VB Svendsen CN Induced pluripotent stem cells: a new revolution for clinical neurology? Lancet Neurol 2011 10 383 394 10.1016/S1474-4422(11)70022-9 21435601

[B12] Farrer M Chan P Chen R Tan L Lincoln S Hernandez D Forno L Gwinn-Hardy K Petrucelli L Hussey J Lewy bodies and parkinsonism in families with parkin mutations Ann Neurol 2001 50 293 300 10.1002/ana.1132 11558785

[B13] Savitt JM Dawson VL Dawson TM Diagnosis and treatment of Parkinson disease: molecules to medicine J Clin Invest 2006 116 1744 1754 10.1172/JCI29178 16823471 PMC1483178

[B14] Ohta S Imaizumi Y Okada Y Akamatsu W Kuwahara R Ohyama M Amagai M Matsuzaki Y Yamanaka S Okano H Kawakami Y Generation of human melanocytes from induced pluripotent stem cells PLoS One 2011 6 e16182 10.1371/journal.pone.0016182 21249204 PMC3020956

[B15] Takahashi K Tanabe K Ohnuki M Narita M Ichisaka T Tomoda K Yamanaka S Induction of pluripotent stem cells from adult human fibroblasts by defined factors Cell 2007 131 861 872 10.1016/j.cell.2007.11.019 18035408

[B16] Matigian N Abrahamsen G Sutharsan R Cook AL Vitale AM Nouwens A Bellette B An J Anderson M Beckhouse AG Disease-specific, neurosphere-derived cells as models for brain disorders Dis Model Mech 2010 3 785 798 10.1242/dmm.005447 20699480

[B17] Nguyen HN Byers B Cord B Shcheglovitov A Byrne J Gujar P Kee K Schule B Dolmetsch RE Langston W LRRK2 mutant iPSC-derived DA neurons demonstrate increased susceptibility to oxidative stress Cell Stem Cell 2011 8 267 280 10.1016/j.stem.2011.01.013 21362567 PMC3578553

[B18] Sies H Glutathione and its role in cellular functions Free Radic Biol Med 1999 27 916 921 10.1016/S0891-5849(99)00177-X 10569624

[B19] Williamson TP Johnson DA Johnson JA Activation of the Nrf2-ARE pathway by siRNA knockdown of Keap1 reduces oxidative stress and provides partial protection from MPTP-mediated neurotoxicity Neurotoxicology 2012 33 272 279 10.1016/j.neuro.2012.01.015 22342405 PMC3521526

[B20] Ramsey CP Glass CA Montgomery MB Lindl KA Ritson GP Chia LA Hamilton RL Chu CT Jordan-Sciutto KL Expression of Nrf2 in neurodegenerative diseases J Neuropathol Exp Neurol 2007 66 75 85 10.1097/nen.0b013e31802d6da9 17204939 PMC2253896

[B21] Tufekci KU Civi Bayin E Genc S Genc K The Nrf2/ARE Pathway: a promising target to counteract mitochondrial dysfunction in parkinson's disease Parkinsons Dis 2011 2011 314082 21403858 10.4061/2011/314082 PMC3049335

[B22] Fukae J Mizuno Y Hattori N Mitochondrial dysfunction in Parkinson's disease Mitochondrion 2007 7 58 62 10.1016/j.mito.2006.12.002 17300997

[B23] Schapira AH Mitochondrial dysfunction in neurodegenerative disorders Biochim Biophys Acta 1998 1366 225 233 10.1016/S0005-2728(98)00115-7 9714816

[B24] Greene JC Whitworth AJ Kuo I Andrews LA Feany MB Pallanck LJ Mitochondrial pathology and apoptotic muscle degeneration in Drosophila parkin mutants Proc Natl Acad Sci USA 2003 100 4078 4083 10.1073/pnas.0737556100 12642658 PMC153051

[B25] Mortiboys H Thomas KJ Koopman WJ Klaffke S Abou-Sleiman P Olpin S Wood NW Willems PH Smeitink JA Cookson MR Bandmann O Mitochondrial function and morphology are impaired in parkin-mutant fibroblasts Ann Neurol 2008 64 555 565 10.1002/ana.21492 19067348 PMC2613566

[B26] Matsuda N Sato S Shiba K Okatsu K Saisho K Gautier CA Sou YS Saiki S Kawajiri S Sato F PINK1 stabilized by mitochondrial depolarization recruits Parkin to damaged mitochondria and activates latent Parkin for mitophagy J Cell Biol 2010 189 211 221 10.1083/jcb.200910140 20404107 PMC2856912

[B27] Narendra D Tanaka A Suen DF Youle RJ Parkin is recruited selectively to impaired mitochondria and promotes their autophagy J Cell Biol 2008 183 795 803 10.1083/jcb.200809125 19029340 PMC2592826

[B28] Tanaka A Parkin-mediated selective mitochondrial autophagy, mitophagy: Parkin purges damaged organelles from the vital mitochondrial network FEBS Lett 2010 584 1386 1392 10.1016/j.febslet.2010.02.060 20188730 PMC2843751

[B29] Yoshii SR Kishi C Ishihara N Mizushima N Parkin mediates proteasome-dependent protein degradation and rupture of the outer mitochondrial membrane J Biol Chem 2011 286 19630 19640 10.1074/jbc.M110.209338 21454557 PMC3103342

[B30] Seibler P Graziotto J Jeong H Simunovic F Klein C Krainc D Mitochondrial Parkin recruitment is impaired in neurons derived from mutant PINK1 induced pluripotent stem cells J Neurosci 2011 31 5970 5976 10.1523/JNEUROSCI.4441-10.2011 21508222 PMC3091622

[B31] Shults CW Lewy bodies Proc Natl Acad Sci USA 2006 103 1661 1668 10.1073/pnas.0509567103 16449387 PMC1413649

[B32] Pramstaller PP Schlossmacher MG Jacques TS Scaravilli F Eskelson C Pepivani I Hedrich K Adel S Gonzales-McNeal M Hilker R Lewy body Parkinson's disease in a large pedigree with 77 Parkin mutation carriers Ann Neurol 2005 58 411 422 10.1002/ana.20587 16130111

[B33] Sasaki S Shirata A Yamane K Iwata M Parkin-positive autosomal recessive juvenile Parkinsonism with alpha-synuclein-positive inclusions Neurology 2004 63 678 682 10.1212/01.WNL.0000134657.25904.0B 15326242

[B34] Schlossmacher MG Frosch MP Gai WP Medina M Sharma N Forno L Ochiishi T Shimura H Sharon R Hattori N Parkin localizes to the Lewy bodies of Parkinson disease and dementia with Lewy bodies Am J Pathol 2002 160 1655 1667 10.1016/S0002-9440(10)61113-3 12000718 PMC1850875

[B35] Chung KK Zhang Y Lim KL Tanaka Y Huang H Gao J Ross CA Dawson VL Dawson TM Parkin ubiquitinates the alpha-synuclein-interacting protein, synphilin-1: implications for Lewy-body formation in Parkinson disease Nat Med 2001 7 1144 1150 10.1038/nm1001-1144 11590439

[B36] Petrucelli L O'Farrell C Lockhart PJ Baptista M Kehoe K Vink L Choi P Wolozin B Farrer M Hardy J Cookson MR Parkin protects against the toxicity associated with mutant alpha-synuclein: proteasome dysfunction selectively affects catecholaminergic neurons Neuron 2002 36 1007 1019 10.1016/S0896-6273(02)01125-X 12495618

[B37] Shimura H Schlossmacher MG Hattori N Frosch MP Trockenbacher A Schneider R Mizuno Y Kosik KS Selkoe DJ Ubiquitination of a new form of alpha-synuclein by parkin from human brain: implications for Parkinson's disease Science (New York, NY) 2001 293 263 269 10.1126/science.1060627 11431533

[B38] Yagi T Kosakai A Ito D Okada Y Akamatsu W Nihei Y Nabetani A Ishikawa F Arai Y Hirose N Establishment of induced pluripotent stem cells from centenarians for neurodegenerative disease research PLoS One 2012 7 e41572 10.1371/journal.pone.0041572 22848530 PMC3405135

[B39] Hargus G Cooper O Deleidi M Levy A Lee K Marlow E Yow A Soldner F Hockemeyer D Hallett PJ Differentiated Parkinson patient-derived induced pluripotent stem cells grow in the adult rodent brain and reduce motor asymmetry in Parkinsonian rats Proc Natl Acad Sci USA 2010 107 15921 15926 10.1073/pnas.1010209107 20798034 PMC2936617

[B40] Park IH Arora N Huo H Maherali N Ahfeldt T Shimamura A Lensch MW Cowan C Hochedlinger K Daley GQ Disease-Specific Induced Pluripotent Stem Cells Cell 2008 10.1016/j.cell.2008.07.041 PMC2633781 18691744

[B41] Soldner F Hockemeyer D Beard C Gao Q Bell GW Cook EG Hargus G Blak A Cooper O Mitalipova M Parkinson's disease patient-derived induced pluripotent stem cells free of viral reprogramming factors Cell 2009 136 964 977 10.1016/j.cell.2009.02.013 19269371 PMC2787236

[B42] Devine MJ Ryten M Vodicka P Thomson AJ Burdon T Houlden H Cavaleri F Nagano M Drummond NJ Taanman JW Parkinson's disease induced pluripotent stem cells with triplication of the alpha-synuclein locus Nat Commun 2011 2 440 21863007 10.1038/ncomms1453 PMC3265381

[B43] Suemori H Yasuchika K Hasegawa K Fujioka T Tsuneyoshi N Nakatsuji N Efficient establishment of human embryonic stem cell lines and long-term maintenance with stable karyotype by enzymatic bulk passage Biochem Biophys Res Commun 2006 345 926 932 10.1016/j.bbrc.2006.04.135 16707099

[B44] Okada Y Matsumoto A Shimazaki T Enoki R Koizumi A Ishii S Itoyama Y Sobue G Okano H Spatiotemporal recapitulation of central nervous system development by murine embryonic stem cell-derived neural stem/progenitor cells Stem cells (Dayton, Ohio) 2008 26 3086 3098 10.1634/stemcells.2008-0293 18757299

[B45] Koike M Shibata M Waguri S Yoshimura K Tanida I Kominami E Gotow T Peters C von Figura K Mizushima N Participation of autophagy in storage of lysosomes in neurons from mouse models of neuronal ceroid-lipofuscinoses (Batten disease) Am J Pathol 2005 167 1713 1728 10.1016/S0002-9440(10)61253-9 16314482 PMC1613187

[B46] Mitsui J Takahashi Y Goto J Tomiyama H Ishikawa S Yoshino H Minami N Smith DI Lesage S Aburatani H Mechanisms of genomic instabilities underlying two common fragile-site-associated loci, PARK2 and DMD, in germ cell and cancer cell lines Am J Hum Genet 2010 87 75 89 10.1016/j.ajhg.2010.06.006 20598272 PMC2896763

