# Supplementary figures and images for: Mitochondrial dysfunction associated with increased oxidative stress and α-synuclein accumulation in PARK2 iPSC-derived neurons and postmortem brain tissue
Source: Mol Brain. 2012 Oct 6;5:35. doi: 10.1186/1756-6606-5-35 (PMC3546866; doi:10.1186/1756-6606-5-35)

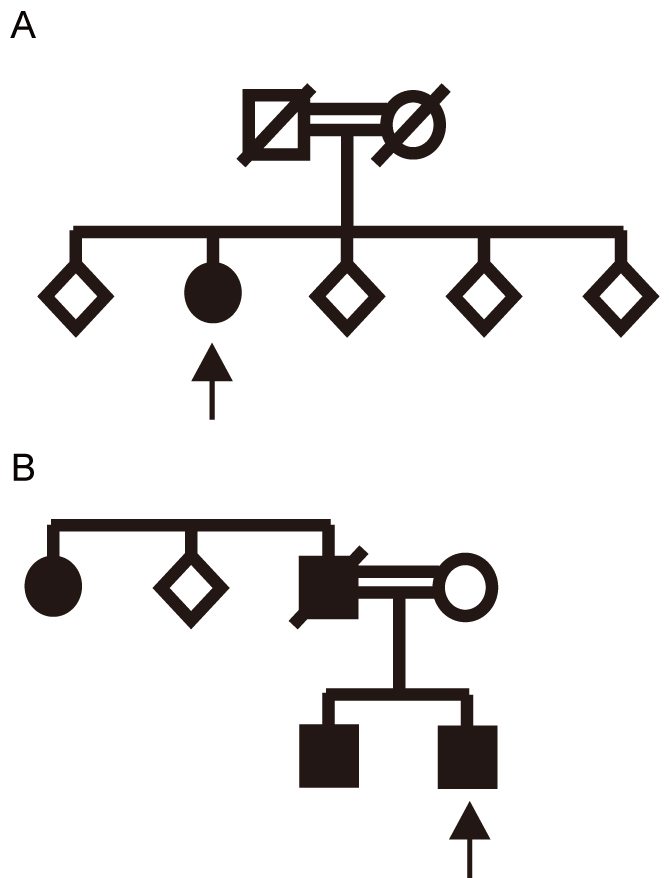

Supplement: Additional file 1 — Genetic studies of family. (A) An arrow indicates PA patient. (B) An arrow indicates PB patient. Filled circles and squares, women and men with PARK2 mutation; Open circles and squares, normal women and men; Diamond shapes, family members whose DNA samples were not analyzed. Symbols with lines through them represent the deceased. [file 1756-6606-5-35-S1.jpeg]

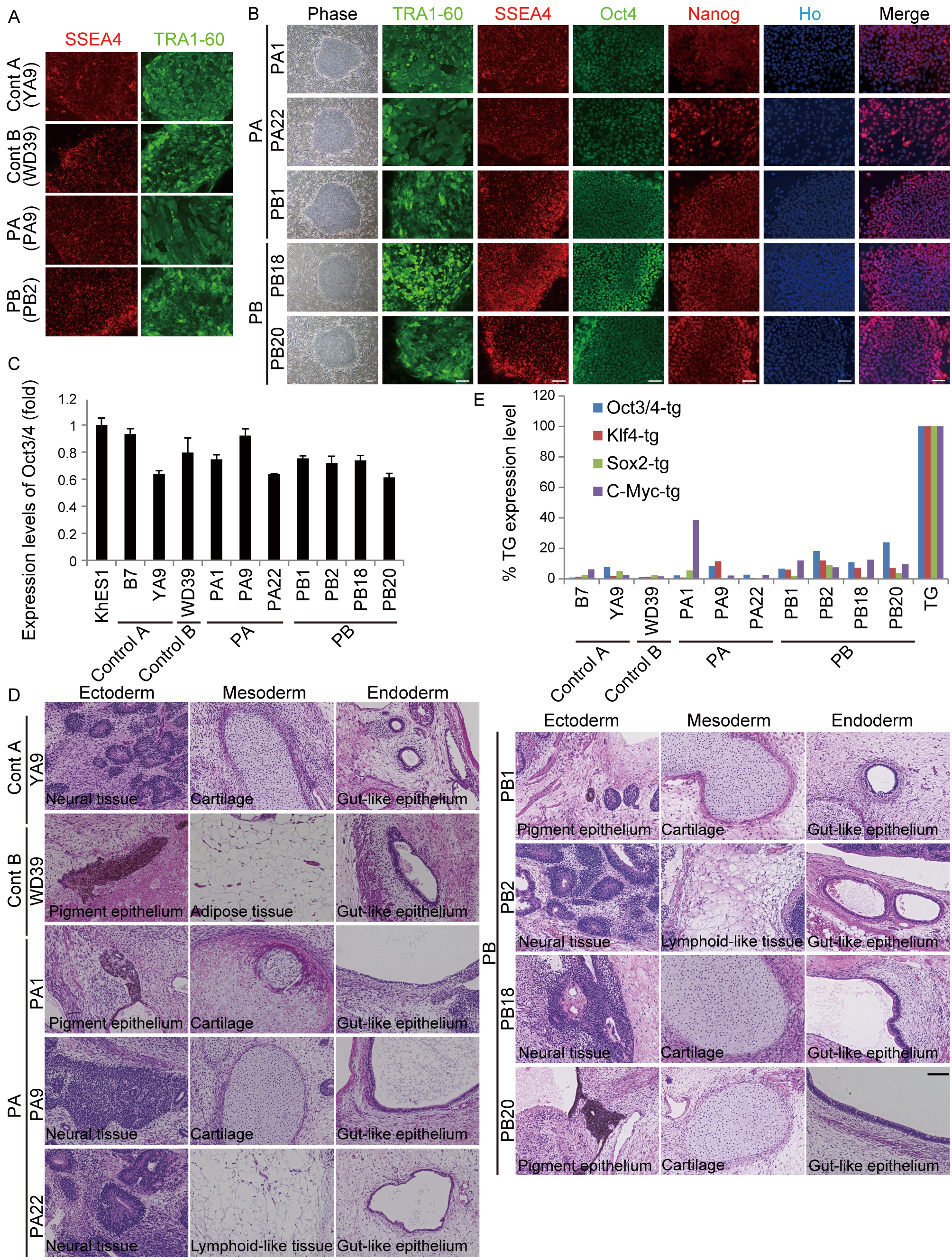

Supplement: Additional file 2 — Characterization of control and PARK2 iPSCs. (A) Control A (YA9), Control B (WD39), PA (PA9), and PB (PB2) iPSCs expressed the pluripotency markers SSEA4 (red) and TRA1-60 (green). Scale bar, 100 μm. (B) iPSCs established from patients PA (PA1, PA22) and PB (PB1, PB18, and PB20) were positive for the pluripotency markers Nanog (red), Oct4 (green), SSEA4 (red), and TRA1-60 (green). Scale bars: phase images, 200 μm; immunofluorescence images, 100 μm. (C) Levels of endogenous Oct4 mRNA in the generated iPSCs were similar to those in KhES1 cells, a human embryonic stem cell (hESC) line [42]. Expression levels were normalized to that of KhES1 (set as 1). (D) Cont A (YA9), Cont B (WD39), PA (PA1, 9 and 22), and PB (PB1, 2, 18 and 20) iPSCs gave rise to teratomas with all three germ layers, confirming pluripotency. Scale bar, 100 μm. (E) Silencing of transgenes in control and PARK2 iPSC clones. Expression levels were normalized to the positive control of fibroblasts in cultures assayed 6 days after retroviral infection ( = 100). Cont A, Control A; Cont B, Control B. [file 1756-6606-5-35-S2.jpeg]

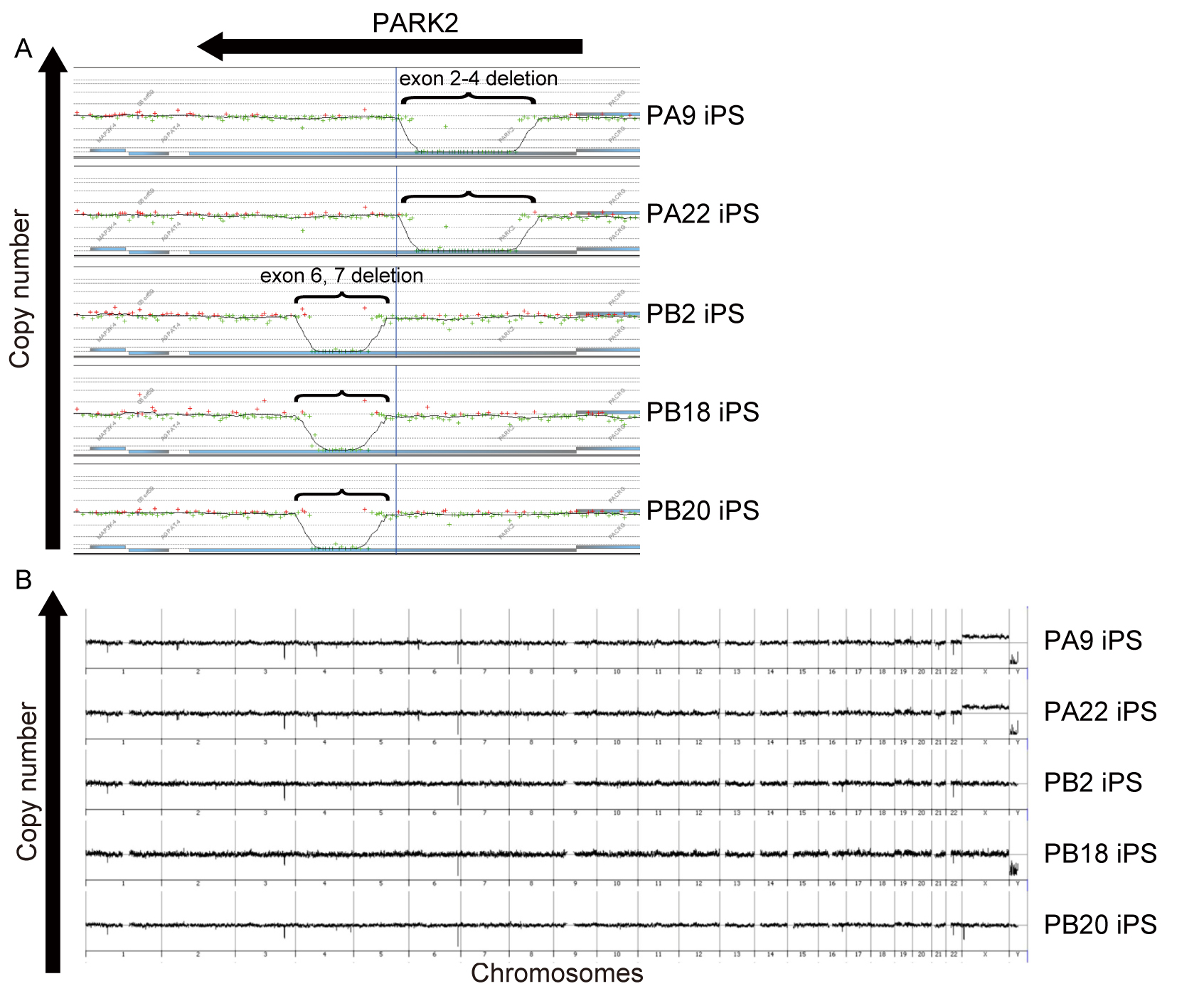

Supplement: Additional file 3 — Confirmation ofparkindeletions and genomic stability of PARK2 iPSCs using comparative genomic hybridization (CGH) microarray analysis. (A) Exons 2–4 were deleted in the PA9 and PA22 iPSC lines. Exons 6 and 7 were deleted in the PB2, 18, and 20 iPSC lines. (B) Copy number profiles of whole chromosomes in PARK2 iPSCs assessed by CGH microarray analysis revealed that no genomic aberrations were introduced during the process of establishing PARK2 iPSCs. [file 1756-6606-5-35-S3.jpeg]

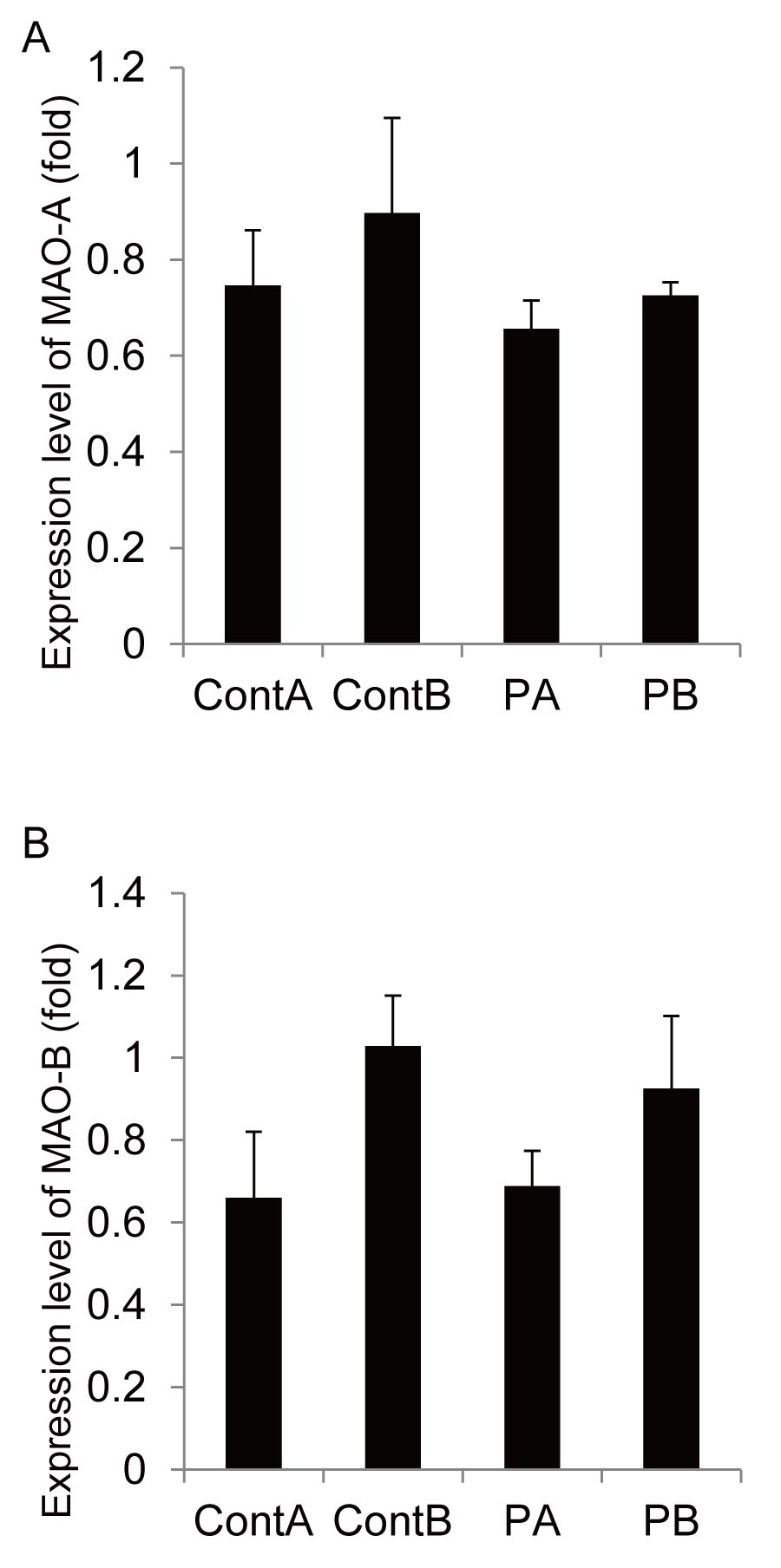

Supplement: Additional file 4 — Expression level of MAO-A and -B showed no difference among Control and PARK2 iPSC-derived neurons. (A,B) qRT-PCR measurement of MAO-A and -B transcripts in PARK2 (PA (1, 9 and 22) and PB (1, 2 and 20)) iPSC-derived neurons showed no difference compared to those in Cont A (B7 and YA9). ContA; Control A, ContB; Control B. [file 1756-6606-5-35-S4.jpeg]

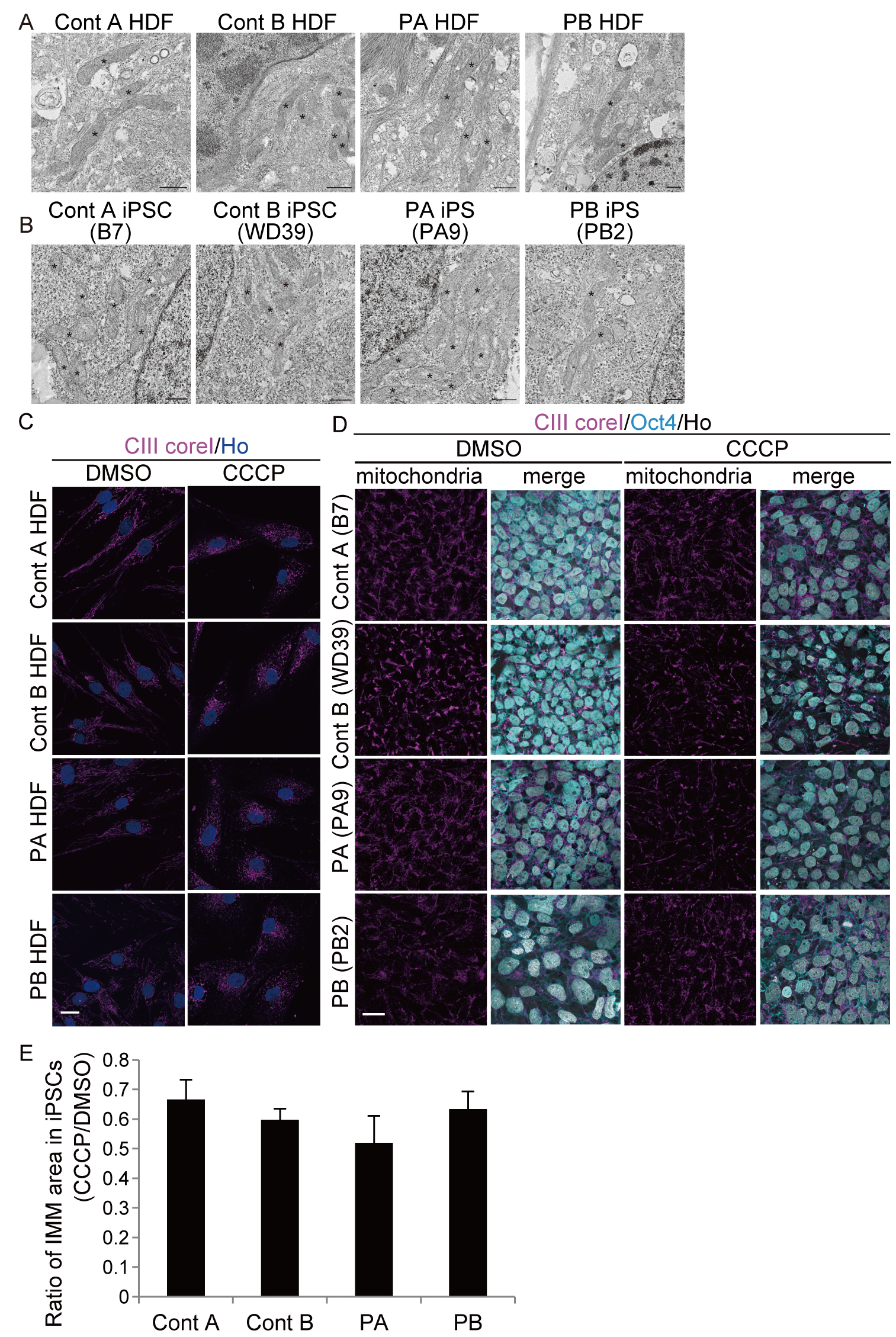

Supplement: Additional file 5 — Healthy mitochondria in PARK2 fibroblasts and iPSCs. (A, B) Electron micrographs of fibroblasts (upper panels) and iPSCs (lower panels) from Control (Cont A and Cont B) and PARK2 patients (PA and PB). Mitochondria in the fibroblasts and iPSCs from both groups showed long, cylindrical profiles with well-organized cristae, and the electron density of the matrix was relatively low (asterisks). Scale bar, 0.25 μm. Cont A, Control A; Cont B, Control B. (C) Fibroblasts were treated with 30μM CCCP or DMSO for 48 h, followed by staining for CIII coreI (magenta) to label the internal mitochondrial membrane (IMM) and counterstaining with Hoechst (Ho, blue). Mitochondrial size decreased after CCCP treatment in both Control (Cont A and Cont B) and PARK2 (PA and PB) fibroblasts. Scale bar, 20 μm. (D) iPSCs were treated with 30 μM CCCP or DMSO for 48 h and then stained for CIII coreI (magenta) to label IMM, Oct4 (blue) to label iPSCs, and Hoechst (Ho, white). Mitochondrial size in Control (Cont A (B7), Cont B (WD39)), and PARK2 (PA9 and PB2) iPSCs decreased after CCCP treatment. Scale bar, 20 μm (E) CCCP/DMSO ratios in Control (Cont A (B7, YA9), Cont B (WD39)), and PARK2 (PA9 and 22 and PB2 and 20) iPSCs (Mann Whitney U-test). Data represent the mean and SEM (n > 3 for each group). [file 1756-6606-5-35-S5.jpeg]

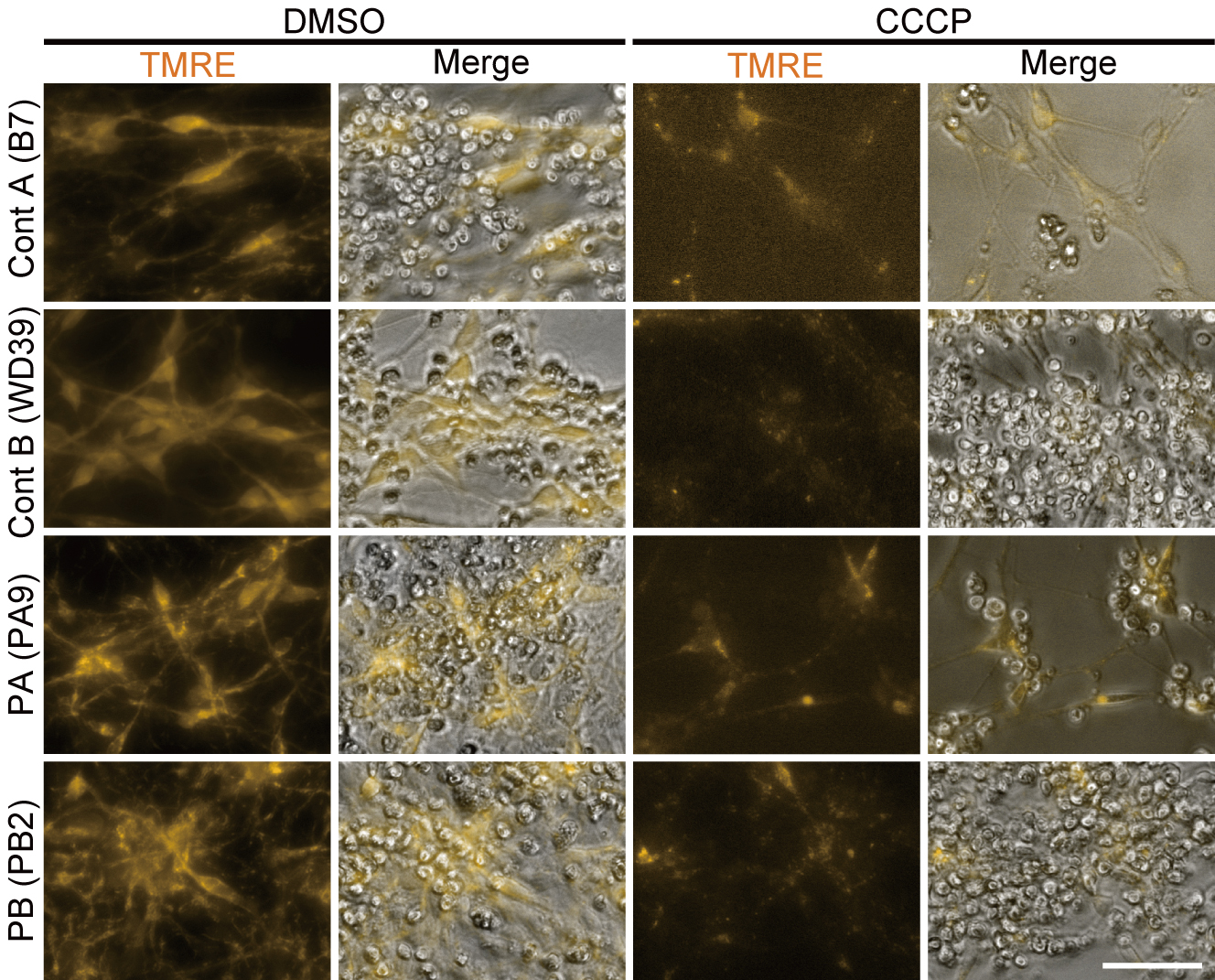

Supplement: Additional file 6 — Mitochondrial membrane potential after CCCP treatment in control and PARK2 iPSC-derived neurons. (A) iPSC-derived neurons were treated with 30 μM CCCP or DMSO for 48 h, after which they were stained for the mitochondrial membrane potential marker, TMRE. The intensity of TMRE (yellow) was clearly reduced in control (Cont A (B7), Cont B (WD39)), and PARK2 (PA9 and PB2) iPSC-derived neurons. Scale bar, 50 μm. [file 1756-6606-5-35-S6.jpeg]

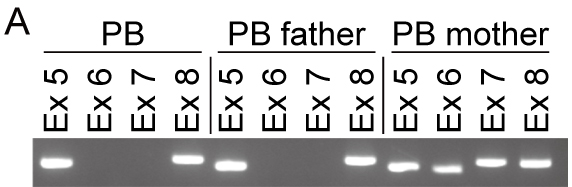

Supplement: Additional file 7 — Confirmation ofparkindeletions carried by the father of patient PB. (A) Deletion of exons 6 and 7 was confirmed in blood samples from PB and the father of PB by PCR. [file 1756-6606-5-35-S7.jpeg]

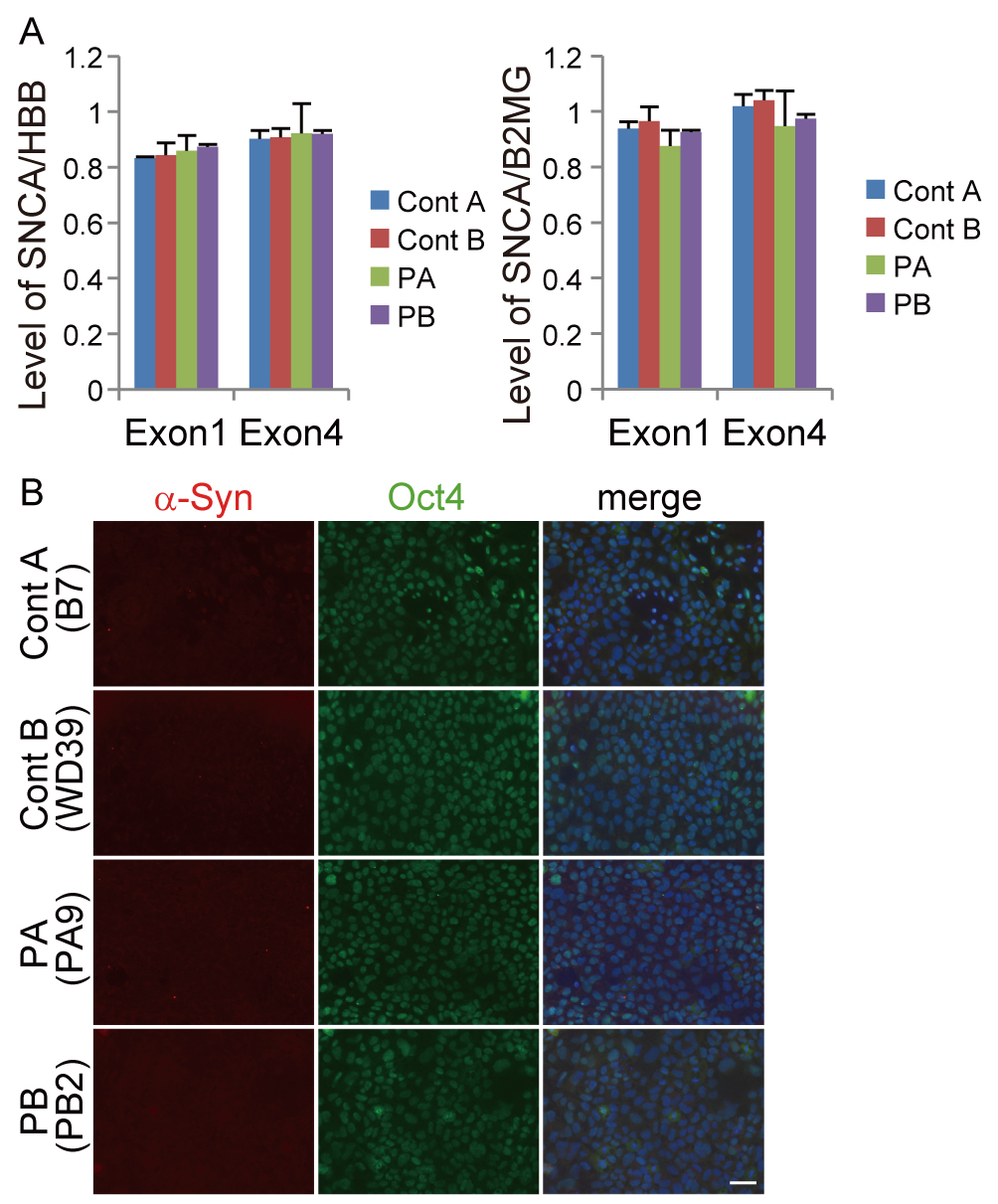

Supplement: Additional file 8 — α-Synuclein signals are not seen in PARK2 iPSCs. (A) Quantitative genomic PCR analysis for SNCA exons 1 and 4 demonstrated a normal copy number in PARK2 (PA1, 9 and 22, and PB1, 2, 18 and 20) iPSCs. The copy number was the same as that observed for Cont A (B7 and YA9) and Cont B (WD39). The SNCA gene copy number was normalized to β-globin (HBB) and β2-microglobulin (B2MG). (B) iPSCs were stained for α-synuclein (red), Oct4 (green; to label iPSCs) and Hoechst (blue). No α-synuclein signals were observed in Cont A (B7 and YA9), Cont B (WD39), or PARK2 (PA9 and 22, PB2 and 20) iPSCs. Scale bar, 50 μm. [file 1756-6606-5-35-S8.jpeg]
